# Pathologic myopia as a concurrent condition in Pierre Robin sequence: a case report and literature review

**DOI:** 10.3389/fmed.2026.1762569

**Published:** 2026-03-13

**Authors:** Yi Liu, Xiao-Yan Zhang, Yuan-Yuan Hu, Ying Wen, Hong-Sheng Bi

**Affiliations:** Ophthalmic Hospital Affiliated to Shandong University of Traditional Chinese Medicine, Jinan, China

**Keywords:** case report, pathologic myopia, syndromic Pierre Robin sequence, posterior scleral reinforcement, Stickler Syndrome

## Abstract

**Introduction:**

Pierre Robin sequence (PRS) is a rare congenital disorder often associated with multisystem abnormalities, yet cases combined with pathologic myopia and medial upper eyelid entropion with trichiasis in whom no pathogenic genetic variants are detectable are rare.

**Case presentation:**

This article reports on a 4-year-old male child with syndromic PRS, whose parents verbally stated that no pathogenic variants were identified in previous genetic testing. Since birth, he has undergone multiple surgeries for micrognathia, cleft palate, and secretory otitis media. He presented for ophthalmic evaluation due to high myopia in both eyes. Ocular examinations revealed the following: best-corrected visual acuity (BCVA) was 0.12 in the right eye and 0.1 in the left eye; bilateral medial upper eyelid entropion with trichiasis was observed, accompanied by rough nasal corneal epithelium; the fundus showed tessellated appearance with Grade A2 macular atrophy. The axial length was 31.38 mm in the right eye and 32.13 mm in the left eye, and optical coherence tomography indicated choroidal thinning. The child underwent bilateral posterior scleral reinforcement (PSR). During the 35-month postoperative follow-up, the child underwent second-stage bilateral upper eyelid entropion correction and trialed low-vision aids and acupuncture. Currently, the child's BCVA is 0.3 in the right eye and 0.12 in the left eye while near vision reaches 0.8 at 10 cm with the use of spectacle- mounted near low-vision aids. Axial length growth has slowed compared to pre-operative rates, The rate of axial length growth has slowed compared to the pre-operative period, and the fundus condition remains stable.

**Conclusion:**

Syndromic PRS can be associated with complex ocular abnormalities. Even in cases where no pathogenic genetic variants are identified, multidisciplinary management is crucial. PSR, entropion correction, and early low vision rehabilitation interventions can effectively improve prognosis. Multidisciplinary collaboration and long-term follow-up are essential components of care.

## 1 Introduction

Pierre Robin sequence (PRS) is a rare congenital disorder characterized by congenital micrognathia, glossoptosis, and upper airway obstruction in neonates, with approximately 73%−90% cases associated with cleft palate (1–3). The global birth prevalence is estimated at about 9.5/100,000 (4). Its pathogenesis is not fully understood and may involve genetic factors, embryonic developmental malformations, and environmental factors. It is classified into isolated PRS and syndromic PRS based on the presence of other systemic malformations, with more than 60% of cases belonging to syndromic PRS (5). Previous literature reports that syndromic PRS is often associated with ophthalmic conditions such as glaucoma, cataract, high myopia, and macular disorders (6–8). Cases with rare ocular manifestations like keratoconus, corneal edema, lacrimal drainage anomalies, and unilateral anophthalmia (9–11) have also been reported. Among these, PRS patients with congenital myopia, vitreous anomalies, and other ocular manifestations, along with pathogenic variants in genes such as COL2A1 and COL11A1, are often diagnosed with Stickler Syndrome (SS) (12). SS is a highly heterogeneous, multisystem hereditary connective tissue disorder caused by collagen abnormalities and is the most common syndrome associated with syndromic PRS (13). Its clinical manifestations can involve the ocular, craniofacial, skeletal, and auditory systems, with high myopia, glaucoma, cataract and vitreoretinopathies being the most common ocular features (14, 15). To date, at least six collagen-encoding genes (COL2A1, COL11A1, COL11A2, COL9A1, COL9A2, COL9A3) have been identified as pathogenic contributors to SS. However, some patients with a clinical diagnosis of SS lack identifiable pathogenic variants on conventional genetic testing (16); this may be attributed to undiscovered disease loci, regulatory variants in non-coding regions, or epigenetic alterations. This study reports on the ophthalmic treatment and follow-up of a child with syndromic PRS associated with bilateral pathologic myopia, entropion and trichiasis, with genetic testing reportedly showing no pathogenic variants.

## 2 Case presentation

**Figure 1:**
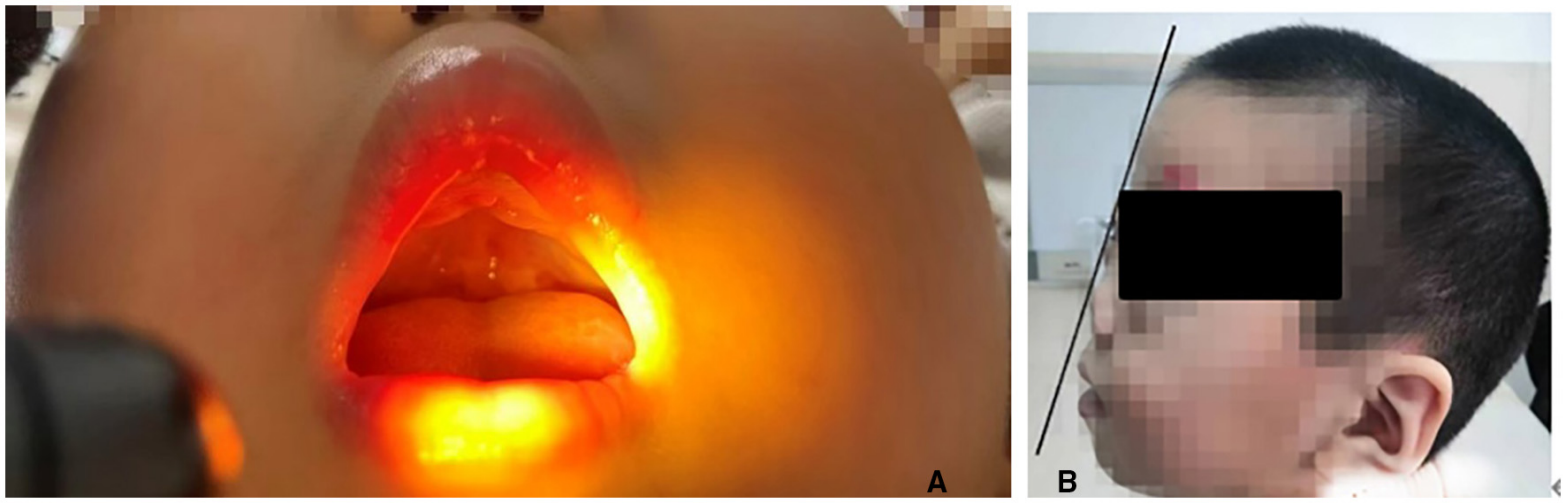
**(A, B)** Craniofacial appearance after surgical treatment: relatively flat facial profile, micrognathia, glossoptosis.

**Figure 2:**
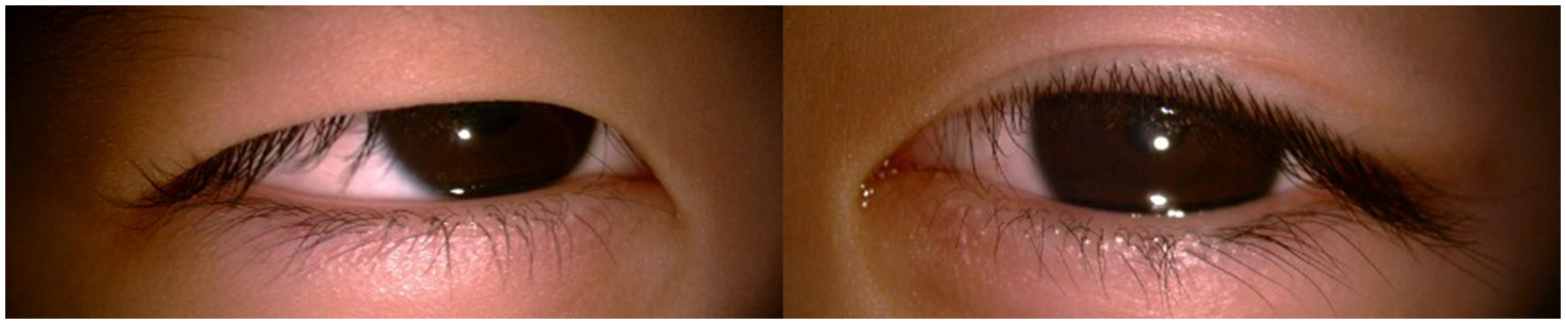
**(A, B)** Bilateral medial upper eyelid entropion and trichiasis.

**Figure 3:**
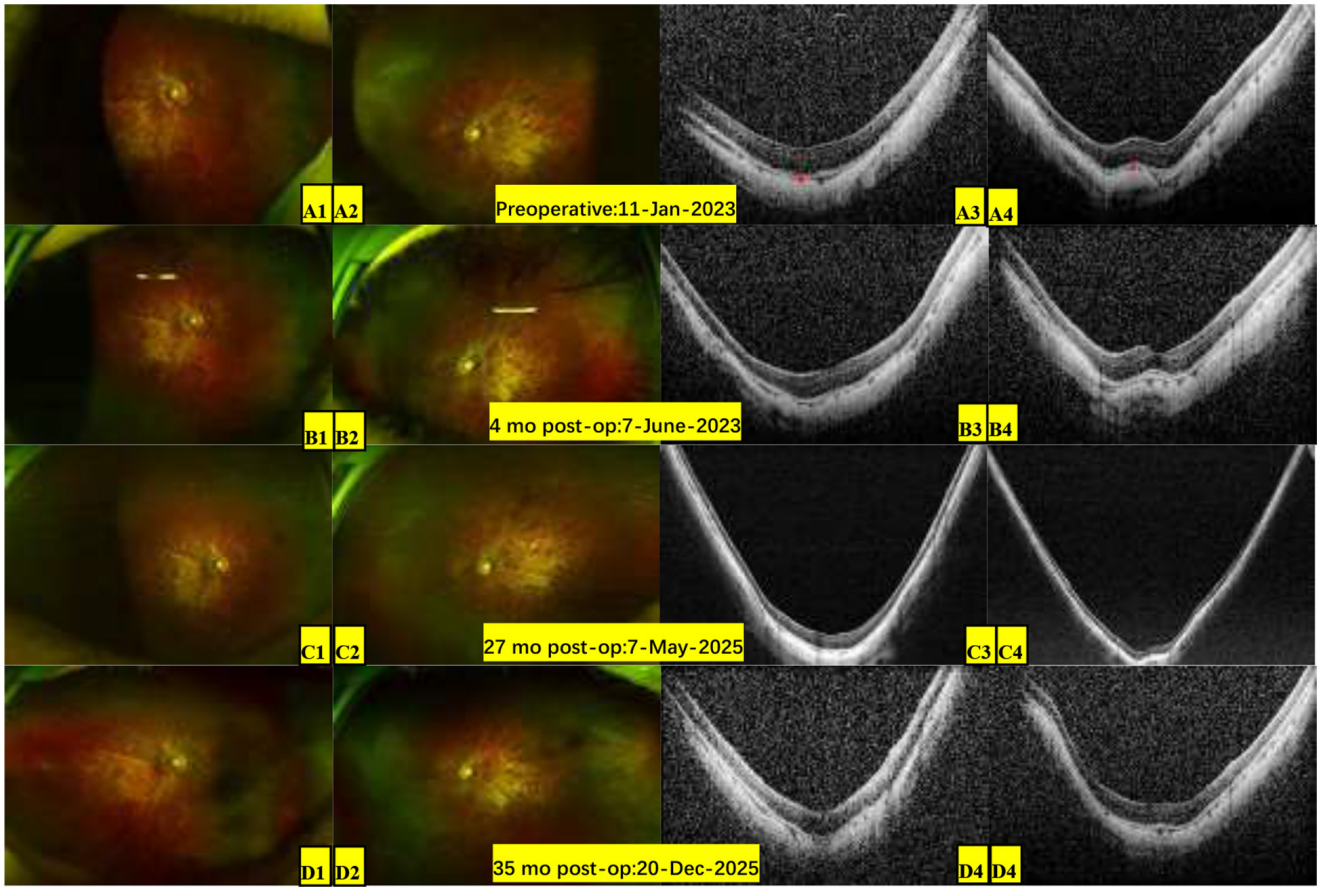
Pre-operative baseline and postoperative bilateral fundus images. **(A)** (Pre-operative): 11-Jan-2023. Ultra-widefield fundus imaging: **(A1)** Right eye and **(A2)** left eye: Tessellated fundus with temporal myopic crescents and diffuse macular atrophy (myopic macular degeneration, Grade **A2**); OCT findings: **(A3)** Right eye and **(A4)** left eye scans reveal choroidal thinning and atrophy (red line), flattened foveal contour, and thinned neurosensory retina with reduced reflectivity. **(B)** (More than 4 months postoperatively): 7-June-2023. **(C)** (More than 27 months postoperatively): 7-May-2025. **(D)** (Around 35 months postoperatively): Dec 20, 2025. **(B–D)** Demonstrate a stable fundus appearance in both eyes, with no progression of the tessellated pattern or macular atrophy over the follow-up compared to the preoperative baseline **(A)**.

**Figure 4:**
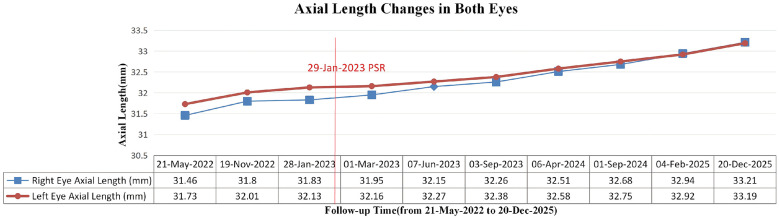
Bilateral axial length changes in a child with syndromic Pierre Robin sequence before and after posterior scleral reinforcement. The red solid line indicates the date of bilateral PSR surgery (29-Jan-2023). Preoperative baseline progression (from 21-May-2022 to 28-Jan-2023): the annual axial length elongation rate was approximately 0.56 mm/year in the right eye and 0.60 mm/year in the left eye; Postoperative progression (from 28-Jan-2023 to 20-Dec-2025): the average annual axial length elongation rate slowed to approximately 0.46 mm/year in the right eye and 0.36 mm/year in the left eye, indicating a slowed and stabilized growth rate postoperatively. Blue line: right eye axial length (mm); red line: left eye axial length (mm).

A 4-year-old male presented to our ophthalmology outpatient department due to bilateral high myopia, with a history of poor binocular vision since infancy, previously having undergone relevant examinations at other hospitals. The child was born via full-term cesarean section with a history of oxygen inhalation at birth. His parents stated that genetic testing had been performed previously with no pathogenic variants detected, and the child was diagnosed with PRS. Since birth, he had undergone multiple surgeries for micrognathia, cleft palate, and secretory otitis media. The parents and an elder sister were healthy, with no family history of genetic disorders. Physical examination of the craniofacial region reveled a relatively flat facial profile, micrognathia, and glossoptosis (Figures 1A, B). Ocular examination: best-corrected visual acuity (BCVA): right eye 0.12, left eye 0.1; intraocular pressure was normal bilaterally. Slit-lamp examination: bilateral medial upper eyelid entropion with eyelashes contacting the cornea (Figures 2A, B); bilateral nasal corneal epithelium roughness, deep anterior chamber, tessellated fundus with temporal crescents, and diffuse macular atrophy (myopic macular degeneration Grade A2) (Figures 3A1, A2). Posterior segment optical coherence tomography (OCT) revealed bilateral choroidal thinning and atrophy, a flattened foveal contour, and a thinned neurosensory retina with reduced reflectivity (Figures 3A3, A4). Axial length: right eye 31.38 mm, left eye 32.13 mm (Figure 4). Corneal topography: right eye thinnest corneal thickness 466 μm, corneal curvature K1: 40.4 D, K2: 42.5 D; left eye thinnest corneal thickness 456 μm, corneal curvature K1: 39.7 D, K2: 42.0 D.

Staged surgical interventions were performed on the child. The first stage involved bilateral posterior scleral reinforcement (PSR). During the 35-month postoperative follow-up, the second-stage bilateral upper eyelid entropion correction was performed, and the child also trialed the use of a personalized spectacle-mounted near low-vision aid (magnification 2.5x, working distance 10 cm) to assist with reading and daily activities. Near visual acuity reached 0.8 at 10 cm, but the aid was not worn regularly. He also received acupuncture treatment (standard acupuncture points: Baihui, bilateral Meichong, Qucha, Cuanzhu, Yuyao, Sizhukong, Sibai, Zusanli, Sanyinjiao, Shen'guan, Jiuli, Taixi, Zhaohai, Shenmai, Taichong, Fenglong, Diji, Fuliu). Due to poor compliance, this did not form a regular treatment course. At the last follow-up, the average annual axial length growth was approximately 0.46 mm for the right eye and 0.36 mm for the left eye (as shown in Figure 4, preoperative annual growth was 0.56 mm for the right eye and 0.60 mm for the left eye; postoperatively, the growth rate slowed and stabilized). BCVA improved to 0.3 in the right eye and remained 0.12 in the left eye, with a combined BCVA binocular was 0.6. The bilateral fundus condition remained essentially stable (as shown in Figures 3B–D, the degree of tessellated fundus and macular atrophy was similar to pre-operative status). No adverse events or other complications occurred during the entire follow-up.

## 3 Discussion

PRS is a rare sequence characterized by congenital micrognathia, glossoptosis, and airway obstruction. Its syndromic phenotype is often accompanied by multisystem developmental abnormalities, necessitating multidisciplinary collaborative management in treatment. Snead et al. (17) proposed that PRS with congenital myopia and cleft palate should raise high suspicion for SS. A retrospective study reported a 47% detection rate of SS among children with syndromic PRS (18). SS is a highly genetically heterogeneous connective tissue disorder encompassing ocular-only, non-ocular, and other subtypes; disease severity is determined by the location and nature of genetic mutations, as well as the role of the corresponding proteins in normal developmental processes, and it constitutes a common cause of pediatric retinal detachment (19). SS exhibits high genetic and phenotypic heterogeneity (20); even within the same family, individuals carrying the same pathogenic variant may present with different clinical manifestation, and there is currently no unified international diagnostic criterion (16). Although there are likely undiscovered genetic phenotypes, molecular genetic testing holds significant value for the diagnosis, prognosis, and management of suspected SS cases (21). The clinical presentation of this child—PRS features, bilateral early-onset pathologic myopia, and otitis media—was highly suggestive of SS. However, out of consideration for the child's long-term privacy protection, the parents refused to provide the previous written genetic testing report and only verbally informed that no pathogenic genetic variants had been detected. Therefore, we recommended further genetic testing to rule out the presence of relevant pathogenic genes and deep intronic mutations (17), yet the parents declined again. We fully respected the family's wishes. Consequently, based on the available clinical information, this study has significant limitations in genetic assessment and cannot definitively confirm whether the child has SS or other related syndromes.

Both syndromic PRS and SS can present with ocular symptoms such as glaucoma, cataract, high myopia, and fundus abnormalities (6–8). Additionally, rare associations such as keratoconus, corneal edema, lacrimal drainage anomalies, and unilateral anophthalmia with syndromic PRS have been reported in sporadic cases (9–11). In clinical practice, the management of the above manifestations is typically symptomatic. Since the prognosis is poor once retinal detachment occurs in SS patients, controlling axial length progression and timely prophylactic retinopexy are crucial management measures for SS patients (16, 22, 23). Huang et al. (24), having systematically compared various myopia control methods including atropine, concluded that PSR is currently the only potentially effective surgical method for the critical objective of slowing axial elongation. Considering this child's young age, although lacking genetic confirmation, his PRS phenotype, early-onset pathologic myopia, and secretory otitis media are highly consistent with the SS phenotypic spectrum, and he exhibited rapid axial length elongation. Based on the prophylactic management principles for SS, we opted for PSR to slow axial length elongation as the primary intervention, rather than pharmaceutical interventions primarily targeting refractive error control, e.g., low-concentration atropine eyedrops (25). Although the clinical efficacy of PSR remains controversial at present, multiple clinical studies in both adults and children with high myopia have confirmed its favorable safety and efficacy in slowing myopia progression and stabilizing vision (26, 27). In this case, after PSR treatment, axial length elongation and fundus structure stabilized (Figures 3, 4) over nearly 3-year of follow-up. This suggests that for such syndromic PRS children with clinical features highly suggestive of SS with rapid axial progression, PSR may be an effective intervention for slowing axial elongation, reducing vitreoretinal traction, and potentially improving choroidal microcirculation (28).

In addition, the child presented with complex anterior segment manifestations, including bilateral entropion and trichiasis—to our knowledge, the first reported instance of these manifestations in both PRS and SS. We speculate that the child's relatively thin central corneal thickness and high astigmatism (approximately 2D) is related to frequent eye rubbing. Studies have indicated that entropion correction performed before the age of 6 is helpful for improving astigmatism (29). At the same time, to prevent further corneal thinning and ectasia induced by trichiasis-related eye rubbing, which may increase the risk of keratoconus (30), we performed bilateral entropion correction for the child. Therefore, monitoring corneal topography changes during follow-up is also an important measure to prevent keratoconus development.

The child is currently in a low-vision state (31), which severely impacts learning ability and quality of life (32). At present, there are no studies on PRS children with low vision. Optical and electronic low-vision aids, along with vision training, have been shown to significantly improve corrected visual acuity (32, 33) and reading speed in children with low vision. In addition, the use of filters, smart devices, and surgical treatments such as miniature telescope implantation have been increasingly applied in the rehabilitation of low-vision patients (34). During follow-up, after trying spectacle-mounted near low-vision aids, the child's near visual acuity reached 0.8 at 10cm, significantly improving functional near vision ability. This underscores the important value of early rehabilitation intervention for enhancing functional vision in preschool children (32, 34). Moreover, this child presents with multiple issues affecting refractive correction, including pathologic myopia, irregular astigmatism, and thin corneal thickness. Scleral Contact Lenses (SCL) may be an ideal tool for addressing such complex refractive problems. SCL can not only correct refractive errors up to −25.00D and irregular astigmatism, but also provide mechanical protection for thin corneas and maintain ocular surface hydration, with some models even exhibiting the effect of slowing myopia progression (35, 36). Although the age for SCL use is routinely considered 12 years and older in clinical practice, the complexity of this case highlights a strong clinical need for future cautious evaluation and potential application of SCL in younger patients.

### 3.1 Limitations

This study has several limitations. First, incomplete genetic assessment evidence is the core limitation, which directly affects the certainty of diagnosis. The child's clinical phenotype is highly consistent with features of SS and other hereditary connective tissue disorders. However, as the parents only provided a verbal report and refused to share written genetic test results, we could not verify the specific technical platform, gene coverage, analysis depth, or ability to detect deep intronic or structural variants of that test (reportedly conducted around 2018). Although we recommended comprehensive genetic sequencing for definitive diagnosis, the family declined again. Therefore, we lack definitive molecular genetic evidence to support or rule out a diagnosis of SS or other syndromes. Consequently, the “syndromic PRS” diagnosis in this report is essentially a clinical description based on phenotype, reflecting the potential gap between clinical phenotype and genetic test results. This limitation also realistically reflects the common ethical and practical challenges faced in clinical research and practice when there is tension between respecting patient autonomy and pursuing precise diagnosis. Furthermore, this is a retrospective single-case report, so the generalizability of its conclusions is limited. Moreover, based on clinical records, subjective information about the patient, such as quality of life and symptom perception, is insufficient in the existing data. During the 35-month follow-up, the child had poor compliance with non-surgical interventions such as low-vision aid wear and acupuncture therapy, making it difficult to systematically evaluate the efficacy of these interventions. Future studies with longer follow-up, larger sample sizes, and a prospective design are needed to validate the multidisciplinary management strategies for PRS children with complex ocular diseases and suspected genetic backgrounds.

## 4 Conclusion

This case report illustrates that a gap may exist between clinical phenotype and genetic test results. Children with syndromic PRS are often diagnosed in pediatrics due to respiratory emergencies at birth, while SS is often first diagnosed in ophthalmology due to common ocular complications. For such children with complex ocular comorbidities, even if genetic testing fails to identify pathogenic variants, multidisciplinary management based on clinical manifestations is still necessary: controlling pathologic myopia progression through PSR, improving corneal status and preventing secondary issues with entropion correction, alongside refractive correction and low vision rehabilitation. Long-term follow-up is crucial for monitoring fundus changes. Although this is a single-case report, it suggests that early personalized intervention is significant for improving visual functional prognosis in children with relevant phenotypes. Furthermore, the value of molecular genetic testing in disease diagnosis, differential diagnosis, and management should be emphasized, and families should be clearly communicated that “no pathogenic gene detected” does not equate to “no genetic risk.”

## Data Availability

The original contributions presented in the study are included in the article/supplementary material, further inquiries can be directed to the corresponding authors.
